# Emerging roles of RNA binding proteins in intervertebral disc degeneration and osteoarthritis

**DOI:** 10.1111/os.13851

**Published:** 2023-10-06

**Authors:** Wen Li, Xing‐Hua Li, Yang Gao, Cheng‐Jie Xiong, Zhong‐Zhi Tang

**Affiliations:** ^1^ Department of Emergency General Hospital of Central Theater Command of PLA Wuhan China; ^2^ Department of Orthopaedic General Hospital of Central Theater Command of PLA Wuhan China

**Keywords:** Intervertebral Disc Degeneration, Osteoarthritis, Post‐Transcriptional Gene Regulation, RNA Binding Proteins

## Abstract

The etiology of intervertebral disc degeneration (IDD) and osteoarthritis (OA) is complex and multifactorial. Both predisposing genes and environmental factors are involved in the pathogenesis of IDD and OA. Moreover, epigenetic modifications affect the development of IDD and OA. Dysregulated phenotypes of nucleus pulposus (NP) cells and OA chondrocytes, including apoptosis, extracellular matrix disruption, inflammation, and angiogenesis, are involved at all developmental stages of IDD and OA. RNA binding proteins (RBPs) have recently been recognized as essential post‐transcriptional regulators of gene expression. RBPs are implicated in many cellular processes, such as proliferation, differentiation, and apoptosis. Recently, several RBPs have been reported to be associated with the pathogenesis of IDD and OA. This review briefly summarizes the current knowledge on the RNA‐regulatory networks controlled by RBPs and their potential roles in the pathogenesis of IDD and OA. These initial findings support the idea that specific modulation of RBPs represents a promising approach for managing IDD and OA.

## Graphical abstract

RNA binding proteins induce RNA life in IDD and OA. RBPs play a central role in PTGR, including transcription, splicing, and export. The effective function of RBPs is critical for coordinating various post‐transcriptional events, and dysfunction of RBPs can lead to apoptosis, extracellular matrix disruption, angiogenesis, and inflammation, which cause IDD and OA.

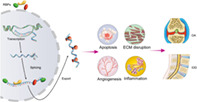

## Introduction

Intervertebral disc degeneration (IDD) and osteoarthritis (OA) are the most common, chronic, and disabling degenerative musculoskeletal disorders. IDD and OA are significant healthcare problems, which cost more than 50 billion dollars per year in US and Canada.^1^, ^2^ IDD causes chronic lower back pain and appears following long‐term undue loading accompanied by multiple pathogenic factors, including aging, smoking, and injury.^3^, ^4^ OA is a major pathogenic cause of knee pain and disability among aging adults. Cartilage destruction and reduction in chondrocytes are the predominant causes of OA, and cartilage plays an essential role in disease pathogenesis.^5^, ^6^ Although IDD and OA exhibit different mechanisms, the co‐pathogenesis is mainly characterized by reduced survival and viability of primary colonizing cells (nucleus pulposus [NP] cells and OA chondrocytes), leading to pathological reconstruction of extracellular matrix (ECM), prolonged inflammatory response, osteophyte formation, and angiogenesis.^7^, ^8^, ^9^ Current therapeutic strategies for IDD and OA are intended to relieve symptoms and minimize pain rather than provide disease‐modifying treatment. Methods for early diagnosis of these degenerative disorders and etiological therapies are lacking. Therefore, a comprehensive understanding of the potential pathogenesis and regulatory mechanisms of IDD and OA is needed.

RNA binding proteins (RBPs) are a large class of regulatory proteins that can bind to target RNA based on specific sequences and form ribonucleoprotein (RNP) complexes.^10^, ^11^, ^12^, ^13^ RBPs affect every aspect of RNA biology, including pre‐mRNA splicing, RNA methylation modification, translation, and degeneration of mRNA. RBPs not only modulate each of these biological processes but also establish a link between them.^14^, ^15^, ^16^ Therefore, RBPs play a central role in post‐transcriptional gene regulation (PTGR). The dynamic interaction between RBPs and their RNA targets is mediated with RNA‐binding domains (RBDs) residing exclusively in RBPs.^13^, ^17^, ^18^ RBDs are deeply conserved across various divergent species and implicated in binding targets and preferences of RBPs.^11^ Consequently, the diversity of RBDs complicates the identification and classification of RBPs. RBPs participate in the biogenesis of ncRNAs and ncRNA‐mediated transcriptional regulation.^19^ Studies have demonstrated that many RBPs cooperate with lncRNAs to regulate post‐transcriptional processes in cellular networks.^20^ Alternatively, ncRNAs can also affect many RBP‐related activities. Previous studies have shown that the biogenesis of RBPs and their target RNAs can also be influenced by circRNAs as super sponges.^21^, ^22^ Multiple studies have demonstrated that ncRNAs play an important role in the pathogenesis of IDD and OA.^6^, ^23^, ^24^

RNA binding proteins are implicated in the regulation of many fundamental cellular processes, such as proliferation, differentiation, and apoptosis.^13^ Dysregulation of RBPs has been demonstrated in a variety of diseases, including cancer,^25^ neurodegenerative disorders,^26^ cardiovascular diseases,^27^ and diabetes.^28^ We speculate that RBPs might hold potential as a biomarker and serve as a novel candidate for therapy design. First, RBPs are abundant and prevalent in various organisms. Second, RBPs are evolutionarily conserved across a variety of divergent species.^29^, ^30^, ^31^ Third, RBPs exhibit high affinity because of the characteristics of RBDs.^32^ Four, RBPs are functionally diverse.^33^, ^34^ Fifth, several studies have found that RBPs are cellular and tissue specific. RBPs are highly expressed in brain and cancer tissues.^35^ Due to recent advancements in RNA‐based technologies,^36^, ^37^, ^38^ increasing evidence has shown that RNA‐regulatory networks controlled by RBPs are also involved in the pathogenesis of IDD and OA.^39^, ^40^, ^41^, ^42^, ^43^, ^44^ IDD and OA are degenerative joint diseases, and RBPs may be involved in the co‐pathogenesis. The RBPs implicated in IDD or OA are summarized in Table 1. Ten of these RBPs are included in this study: HuR, TTP, FUS, YTHDF2, YTHDC2, KRT18, GNL3, CIRBP, RBPMS, and RBMS3. Expression or functional changes of these RBPs disrupt RNA‐regulatory networks controlled by RBPs and play an important role in the pathogenesis of IDD and OA.

**Table 1:** Functional characterization of RBPs in OA and IDD

RBPs	Target DNA or mRNA	Function/Phenotype	Main findings	Reference
In OA				
HuR	NLRP3, COX‐2, NKRF, SOX9	Stability	Regulates inflammation; promotes cell autophagy; maintains cartilage ECM	39
TTP	HSPA1A	Stability	Enhances HSPA1A mRNA decay and inhibits chondrocyte apoptosis	79
FUS	Col10a1, SLC7A2, PDE4B	Transcription, splicing	Regulates chondrocyte hypertrophic differentiation; inhibits the miR‐4498/TIMP3 axis and inflammatory response; regulates chondrocyte cell viability	139
YTHDF2	ATG7	Destabilizing	METTL3‐mediated m^6^A modification induces the decay of the ATG7 transcript in a YTHDF2‐dependent manner	88
YTHDC2	METTL3	RNA binding	Regulates the inflammatory effect of ECM degradation	94
KRT18	–	–	–	–
GNL3	IL‐24, PTN	Genomic regulation	Induces articular osteocyte apoptosis and angiogenesis	25,42
CIRBP	/	Pro‐inflammatory cytokine	CIRP levels are closely associated with the severity of knee OA	43
RBPMS	Smad2/3	Transcription	Forms a counter‐regulatory mechanism with TGF‐β and IL‐1β to maintain the homeostasis of normal articular cartilage	44,140
RBMS3	–	–	–	–
In IDD				
HuR	NF‐κB, ATG7	NP senescence	Suppresses inflammation and promotes ECM homeostasis via NKRF; Prompts Atg7 mRNA stability via binding to ARE	40,62
TTP	TNF‐α	Destabilizing	Anti‐inflammation	29
FUS	Circ‐GRB10	Stability	Inhibits NP cell apoptosis; Circ‐GRB10/miR‐328‐5p/ERBB2 signaling pathway is involved in IDD development	23,85
YTHDF2	FIP200	Destabilizing	Autophagy inhibition; protects FIP200 mRNA from YTHDF2‐mediated degradation	41
YTHDC2	–	–	–	–
KRT18	KRT8	ECM degradation	Cellular structural integrity and possibly signaling	110
GNL3	–	–	–	–
CIRBP	–	–	–	–
RBPMS	–	–	–	–
RBMS3	beta‐catenin	NP cell proliferation	Reduces the expression of β‐catenin and c‐myc in NP cells and inhibits the activity of the Wnt/β‐catenin signaling pathway	150

Abbreviations: ECM, extracellular matrix; IDD, intervertebral disc degeneration; NP, nucleus pulposus; OA, osteoarthritis; RBP, RNA binding protein.

First, we will discuss the disruption of RNA‐regulatory mechanisms regulated by RBPs in IDD and OA (some RNA‐regulatory mechanisms are summarized in Table 1). Second, we will clarify the contribution of the dysregulation induced by RBPs to the pathogenesis of IDD and OA. Finally, we will review how target RBPs have been applied to treat human diseases and discuss their therapeutic potential in treating IDD and OA.

In this review, Medline, Current Contents, the Cochrane Library, Google Scholar, and PubMed were searched without date restriction using a wide range of search terms: “RNA binding protein,” “RNA binding proteins,” “RNA‐binding protein,” “RNA‐binding proteins,” “RBPs,” “RBP” AND “intervertebral disc degeneration,” “IVD,” “IDD,” “IVDD” AND “osteoarthritis,” and “OA.” Only English‐language references were included in this review. Although the focus was on work published in the past 5 years, work frequently referenced and included in highly respected publications older than 5 years was also included.

## Potential role of RNA binding proteins in intervertebral disc degeneration and osteoarthritis

Intervertebral disc degeneration and OA are degenerative diseases of the connecting tissues (intervertebral disc or synovial joint) adjoining bones in which structural integrity is damaged and biomechanical function is impaired. The intervertebral disc (IVD) is a fibro‐cartilaginous structure that transmits loads between adjacent vertebrae and maintains the flexibility and stability of the spine.^45^, ^46^ The synovial joint mainly comprises articular cartilage, synovial fluid, and surrounding soft tissue.^47^, ^48^ IVD and synovial joints share similar structures and functions. IVD and synovial joints are important components of the musculoskeletal system, and they play an essential role in maintaining the mobility and flexibility of the musculoskeletal system.^49^ IVDs and synovial joints experience hundreds of thousands of mechanical loading cycles during a person's lifetime. Accumulated injuries combined with increasing age lead to IDD and OA.^50^, ^51^, ^52^ Environmental and genetic factors are involved in the etiology of both musculoskeletal disorders. Genetic factors have a significant impact on susceptibility and occurrence of IDD and OA.^52^, ^53^

The cellular metabolism in IVD and articular cartilage is a coordinated network dynamically modulated at various levels by external stimuli (environmental factors) and resource availability (genetic factors). Although the molecular mechanisms underlying cellular metabolism remain largely unclear, it is widely accepted that apoptosis, ECM disruption, inflammation, and angiogenesis are involved in the development of IDD and OA.^8^, ^41^, ^54^, ^55^, ^56^ Moreover, studies have demonstrated that these biological processes mentioned above are closely associated with PTGR, which is coordianted by RBPs and ribonucleoprotein (RNP) complexes.^10^, ^11^, ^13^ Multiple studies have demonstrated that ncRNAs regulate phenotypic gene expression and maintain matrix homeostasis in IDD and OA through the post‐transcriptional regulation mechanism.^57^, ^58^ The interaction between RBPs and ncRNAs is critical for PTGR. Moreover, as an addtional layer of post‐transcriptional control, alternative splicing in RBPs has causal effects on ncRNA metabolism.^19^ However, the underlying mechanism of this interaction remains largely unknown in IDD and OA. Therefore, RBP might play an essential role in the pathogenesis of IDD and OA.

**Fig. 1:**
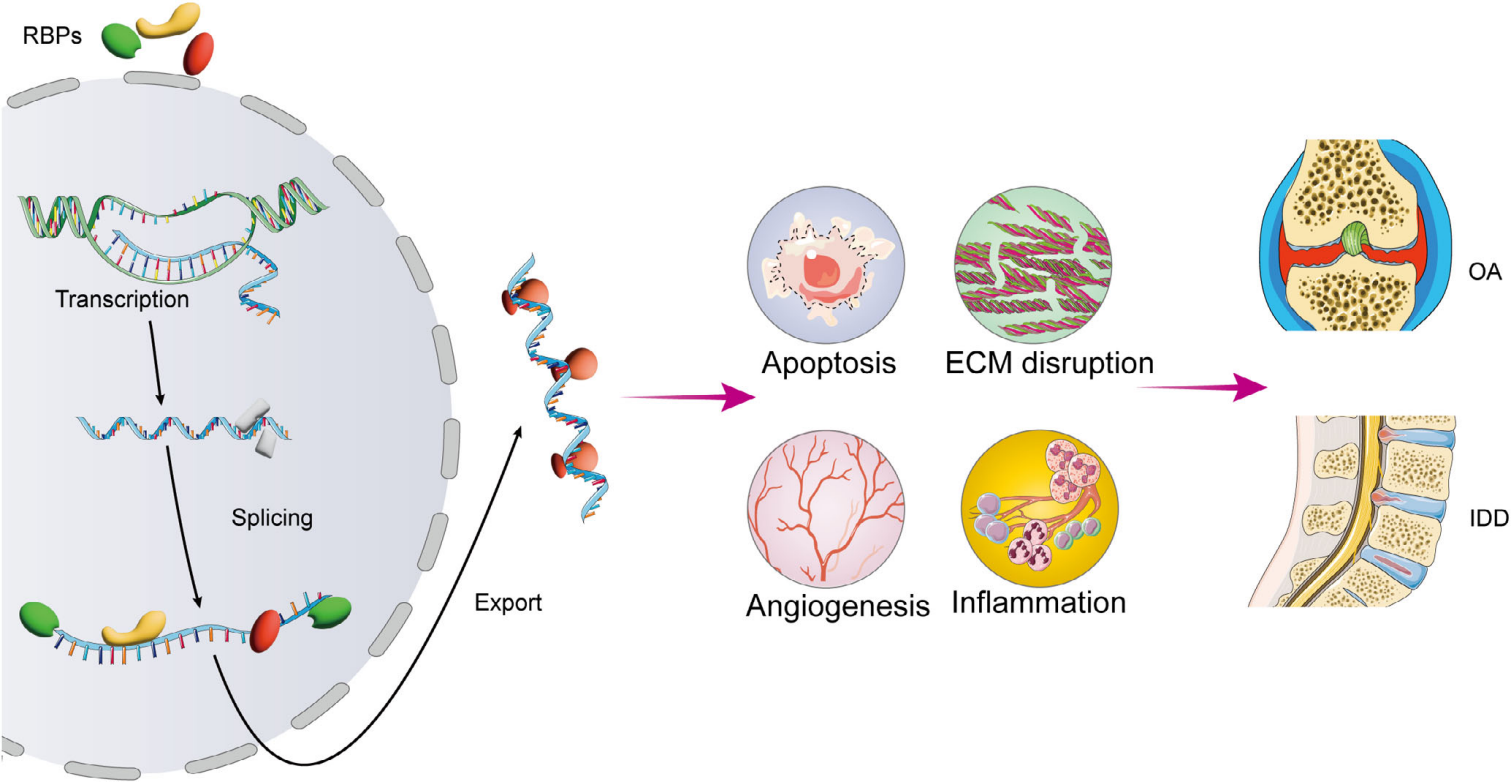
RNA binding proteins (RBPs) induce RNA life in intervertebral disc degeneration (IDD) and osteoarthritis (OA). RBPs play a central role in PTGR, including transcription, splicing, and export. Appropriate function of RBPs is critical for coordinating various post‐transcriptional events, and dysfunction of RBPs might lead to apoptosis, extracellular matrix (ECM) disruption, angiogenesis, and inflammation, which cause IDD and OA.

Figure 1 presents RBPs that contribute to pathogenic events triggered in IDD and OA. We will discuss three aspects in this section: the roles of specific RBPs (HuR, TTP, FUS, YTHDF2, YTHDC2, KRT18, GNL3, CIRBP, RBPMS, and RBMS3) in RNA metabolism; dysregulation of these RBPs in IDD and OA; and their contribution to the development of IDD and OA.

### Elav‐like protein 1 (Elavl1) (HuR)

HuR is a multi‐functional RBP that belongs to the embryonic lethal abnormal vision‐like (ELAV) protein family.^59^, ^60^ It has been implicated in the pathogenesis of IDD and OA.^39^, ^40^, ^61^, ^62^ HuR has been reported to be involved in the regulation of mRNA translation and stability.^59^, ^60^ HuR binds to specific mRNAs and transports bound mRNAs from the nucleus to the cytoplasm. It binds to 3′UTR AU‐rich elements (AREs) at the 3′UTRs and regulates the stability of AREs containing mRNA. HuR is a ubiquitously expressed protein that has been widely investigated in cancer.^63^ HuR plays an important role in other diseases, such as cardiovascular disease, neurodegenerative disorders, and diabetes.^64^, ^65^ It is an important post‐transcriptional gene regulator involved in many biological processes, including inflammation, apoptosis, stress response, cell cycle, and immune response.^59^, ^60^

First, recent studies have demonstrated that the expression of HuR is involved in PTGR during IDD and OA through multiple distinct mechanisms.^39^, ^40^, ^61^, ^62^ First, the regulation of degeneration and synthesis of ECM factors plays an essential role in maintaining the homeostasis of articular cartilage.^39^ The ECM homeostasis might be disrupted, and the degeneration exceeds the synthesis of ECM, contributing to the development of OA. Articular cartilage ECM is mainly composed of collagen type II (Col II) and aggrecan (ACAN) regulated by synthesis factor SOX9.^66^, ^67^ In contrast, the degeneration factor matrix metalloproteinase‐13 (MMP‐13) has been demonstrated to break down Col II, a major component of articular cartilage ECM.^68^, ^69^ MMP‐13 is an ARE‐containing mRNA associated with the PTGR by HuR.^39^, ^70^ Knockdown of HuR is associated with a decreased level of MMP‐13. However, HuR might not regulate MMP‐13 through the mRNA stability regulation mechanism; alternatively, it inhibits the expression of MMP‐13 suppressers, including RUNX2, SP1, and USF1. Binding interaction between HuR and the other ARE‐containing mRNA SOX9 was not observed in this study.^39^ The data presented here demonstrated that HuR is involved in the regulation of homoeostasis of articular cartilage ECM.

Second, HuR is also involved in the post‐transcriptional gene regulation during the development of intervertebral disc degeneration.^40^, ^61^, ^62^ Pan et al. demonstrated that HuR also plays a protective role in IDD by maintaining ECM and pH homoeostasis in NP.^61^ However, they did not find that HuR binds to well‐known target mRNA (HIF and VEGF) in NP cells; alternatively, HuR might act as a regulator of many ECM‐related mRNAs, including several collagens, ACAN, MMP‐13, and Ca12 (pH related mRNA). It is plausible that tissue or cellular‐specific PTFR of HuR might impact the ability of HuR to bind specific mRNA in NP cells. Similarly, Shao et al. found that HuR is important for maintaining ECM homeostasis in NP cells.^40^ HuR binds to AREs of NKRF mRNA and promotes its stability, which upregulates the expression of NKRF mRNA and inhibits NF‐kB signaling pathway‐induced inflammation. However, they found that the role of HuR in IDD is complicated. Overexpression of HuR cannot alleviate pathological changes of IDD, whereas knockdown of HuR might increase the sensitivity of NP cells to the proinflammatory cytokine TNF‐α. Taken together, HuR could have implications for the regulation of ECM homoeostasis and inflammation during IDD.

Finally, further studies have found that HuR plays an important role in IDD combined with diabetes through autophagy regulation.^62^ Recent studies have found that PTGR of HuR is related to autophagy;^71^ however, the autophagy regulation of HuR in IDD combined with diabetes remains unknown.^72^ HuR prompts Atg7 mRNA stability by binding to the ARE, resulting in suppression of senescence through autophagy activation. In summary, HuR has an important role in the metabolism regulation of NP cells and chondrocytes, and changes in HuR expression contribute to pathogenic events, including ECM disruption, inflammation and autophagy in OA or IDD.

### ZFP36 (tristetraprolin)

Tristetraprolin (TTP) belongs to the ZFP36 family, consisting of ZFP36 (TTP, TIS11), ZFP36L2 (BRF‐2, TIS11d), ZFP36L1 (BRF‐1, TIS11b), and ZFP36L3.^73^, ^74^ Although members of the ZFP36 family share a similar structure, they might mediate distinct cellular functions according to the cell or tissue type and the nature of the extracellular stimulus.^75^ TTP is a zinc finger protein that contains tandem zinc‐binding motifs, triggering ARE‐containing mRNA destabilization.^76^, ^77^ Like HuR, TTP is a ubiquitously expressed protein that has been extensively investigated in cancer,^78^neurodegenerative disorders,^78^ and diabetes.^79^ Recent studies have shown that TTP is implicated in the pathogenesis of IDD and OA.^39^, ^79^

Several diverse mechanisms have been reported.^80^ First, TTP is involved in regulating homoeostasis of articular cartilage ECM.^82^ Both SOX9 and MMP‐13 are ARE‐containing mRNAs, and an imbalance between them might be due to changes in protein levels caused by the activities of RBPs. TTP is a destabilizing factor that acts as a suppressor of SOX9 expression. TTP binds to AREs of SOX9 mRNA and affects its stability, which downregulates the expression of SOX9 mRNA.^39^, ^70^ Therefore, the knockdown of TTP can increase the expression of SOX9 mRNA. Second, Son et al. found that TTP expression was significantly increased in OA cartilage and chondrocytes compared with normal controls.^79^ TTP encoding HSPA1A belongs to the heat‐shock protein 70 family, which exerts protective effects against apoptosis, inflammation, and oxidative stress in various cell types. TTP might exhibit protective effects on OA pathogenesis by increasing the expression of HSPA1A and inhibiting apoptosis of chondrocytes.

Tristetraprolin has an important role in the metabolism regulation of chondrocytes that contribute to OA pathogenesis. TTP might be a good target for treatment options because inhibition of TTP can exert protective effects on OA by modulating chondrocyte homoeostasis and suppressing inflammation and apoptosis of chondrocytes. However, to our knowledge, the potential role of TTP in IDD has not been reported in the literature.

### Fused in sarcoma

Fused in sarcoma (FUS) is an hnRNP protein that belongs to the FET family, including FUS, EWS, and TAF15.^81^, ^82^ FUS has been extensively studied in neurodegenerative diseases over the past decade, such as amyotrophic lateral sclerosis (ALS) and frontotemporal lobar degeneration.^82^ The structure of FUS consists of an N‐terminal domain with a QGSY‐rich region, a zinc finger motif, and multiple repeated RGG amino acids at the C‐terminal. Various activities of FUS are mediated by both protein–protein and protein–RNA interactions at the post‐transcriptional level. The FUS protein has been reported to be involved in various biological processes, including alternative splicing, RNA transport, mRNA synthesis, and the selection of polyadenylation sites.^81^, ^82^ FUS is mainly located in the nucleus, and a minority is found in the cytoplasm, including neuronal axons. Using RNA immunoprecipitation (RNA‐IP), Colombrita et al. found that hundreds of mRNAs in the neuronal cytoplasm are associated with FUS, and FUS binding sites are located in 3′UTRs of these mRNA.^83^, ^84^ Dysfunction of FUS might impair metabolic pathways of multiple RNA and eventually damage multiple RNA metabolic pathways, eventually leading to neurodegenerative disease. Recently, the possible role of FUS has also been described in other diseases.

Research has demonstrated that FUS plays a role in post‐transcriptional gene regulation during the development of IDD. CircRNAs have been reported to play an important role in IDD.^5^ Guo et al. found that decreased expression of circ‐GRB10 might contribute to IDD pathogenesis by increasing miR‐328‐5p regulated proliferation‐related mRNA through the ErbB pathway.^85^ However, the underlying mechanism regulating circ‐GRB10 synthesis remains unknown. The interaction between RBPs and ncRNAs has been implicated in PTGR. Therefore, Guo et al. demonstrated that FUS binds to specific binding sequences on flanking introns of circ‐GRB10 pre‐mRNA and is involved in circ‐GRB10 pre‐mRNA splicing.^23^ Knockdown of FUS significantly reduces circ‐GRB10 levels and has protective effects on IDD pathogenesis by increasing the proliferation of NP cells. Interestingly, the biogenesis of FUS is controlled by another CircRNAs, miR‐141‐3p. These results suggest that RBP‐associated PTGR plays a ubiquitous role in maintaining NP ECM homeostasis, and FUS and FUS‐regulated circ‐GRB10 might be potential targets in the future treatment of IDD.

### YTHDF2 and YTHDC2

m^6^A is a well‐known PTGR associated with the development of multiple diseases.^86^, ^87^, ^88^, ^89^, ^90^ “Writers,” “erasers,” and “readers” are involved in the basic biology of m^6^A modification. Theler et al. first demonstrated that YTH domain‐containing protein might act as m^6^A readers and interact with m^6^A through a hydrophobic and pyramid‐shaped cage, which comprises two or three tryptophan residues.^91^ There are five members in the YTH domain‐containing protein family: YTHDF1, YTHDF2, YTHDF3, YTHDC1, and YTHDC2.^92^ These YTH domain‐containing RBPs can modulate m^6^A in a structural switch manner.^92^, ^93^, ^94^, ^95^ m^6^A can change the structure of RNA substrates and then trigger the binding of specific YTH domain‐containing RBPs to these RNA substrates, involving a variety of bioprocesses, such as splicing, mRNA stability, localization, and translation.^93^, ^94^ Recently, both YTHDF2 and YTHDC2 have been reported to be associated with the m^6^A modification in the IDD and OA pathogenesis.^41^, ^90^, ^96^

Autophagy is a complicated bioprocess essential for maintaining the survival and health of various cells under unfavorable external stimuli.^97^ Autophagy is associated with multiple human pathologies, including cancer,^98^ neurodegeneration,^99^ and cardiomyopathic disorders.^100^ Recent studies have shown that autophagy is involved in the regulation of apoptosis and reduces the apoptosis rate of NPs.^101^, ^102^ FIP200 is important for the initiating process of autophagy, and the m^6^A “eraser” ALKBH5 mediates m^6^A demethylation of FIP200 mRNA and reduces the apoptosis rate of NPs under compression.^41^, ^102^ The m^6^A “reader” YTHDF2 is involved in this methylation regulation mechanism. Low levels of m^6^A of FIP200 are associated with decreased YTHDF2‐mediated degeneration of FIP200 mRNA.^41^ m^6^A methylation might also play an essential role during the development of OA. Sang et al. found that METTL3 and YTHDC2 were significantly reduced in the knee tissue of AO patients compared with non‐OA patients.^96^ However, their conclusion was mainly based on bioinformatic analysis and cellular experiments. Further study was not conducted to investigate the underlying mechanism of METTL3 and YTHDC2 in the regulation of m^6^A methylation. In addition, several other RBPs have been reported to be associated with the m^6^A methylation regulation mechanism during the development of IDD and OA. Bioinformatic research has shown that the binding of FTO and ZFP217 can lead to the demethylation of LOC102555094 and activation of the downstream of the Wnt pathway, contributing to the dysfunction of glucose metabolism in NP cells.^103^, ^104^

The clarification of the underlying m^6^A methylation regulation mechanism under the control of these RBPs might contribute to developing a method for maintaining cellular homeostasis in response to unfavorable external stimuli. YTHDF2 and YTHDC2 are potential future therapeutic targets for IDD and OA.

### KRT18

KRT18, a main component of intermediate filaments (IF) in simple epithelial cells, undergoes caspase‐mediated cleavage upon epithelial cell necrosis and apoptosis.^105^, ^106^ KRT18 belongs to the human epithelia KRT family consisting of 17 type I and 20 type II members, mainly expressed in epithelial tissues.^106^ With the increasing knowledge of KRT18, dysfunction of KRT18 has also been proven to exist and vary widely in various tumors in a cellular and tissue‐specific manner.^105^, ^107^ Moreover, studies have demonstrated that KRT18 is aberrantly expressed in a variety of human tumors and closely related to clinical progression and prognosis.^105^, ^108^

Rodrigues‐Pinto *et al*. (2017) showed that KRT8, KRT18, and KRT19 are expressed in NP and should be regarded as markers of notochord cells.^109^ In addition, the decreased expression of KRT‐8 and KRT‐18 is closely related to IVD degeneration.^110^, ^111^ This suggests that KRT‐8 and KRT‐18 are also markers of a healthier NP phenotype. Moreover, studies have revealed that KRT18 is not only a biomarker but also a regulator in many diseases.^105^, ^107^, ^108^, ^112^ KRT18, as a multiple‐function RBP, has been involved in various bioprocesses, including cell functions (e.g., cell migration, proliferation, and differentiation).^105^, ^106^, ^107^ Several studies have demonstrated that KRT‐18 regulates cell proliferation and apoptosis through the PTGR mechanism, and it might be a cellular death biomarker.^113^, ^114^, ^115^ However, the specific role of KRT18 within the NP has not been fully characterized to date. Our preliminary results have shown that KRT18 is associated with the apoptosis of NP cells and the regulation of ECM‐related mRNA (data not published). We speculate that KRT18 might be involved in the pathogenesis of impaired NP cell metabolism.

### Nucleolar GTP‐binding protein 3

GTP‐binding protein 3 (GNL3), known as nucleostemin, also plays an important role in PTGR involved in different stages of RNA metabolism. It has been implicated in multiple bioprocesses, including cell proliferation, differentiation, and cell cycle regulation.^116^, ^117^, ^118^ GNL3 belongs to the GTP‐binding protein family, containing an MMRHSR1 domain.^119^ GNL3 is abundantly expressed in bone marrow mesenchymal stem cells (BM‐MSCs) and is closely related to the differentiation of chondrocytes.^116^, ^117^, ^118^ Recently, both human and animal studies found that GNL3 were significantly reduced in knee tissues of OA samples compared with non‐OA samples. However, the potential mechanism underlying aberrant GNL3 expression in OA remains unclear. Several studies have proposed that GNL3 might interact with OA potential susceptibility genes at the post‐transcriptional level and affect the metabolism and phenotypes of chondrocytes during the development of OA.^42^ Zhu et al. found that increased expression of GNL3 can upregulate the expression of IL‐24 and PTN, which has been previously reported as associated with OA.^42^, ^120^, ^121^ IL‐24 and PTN play essential roles in aggravating the inflammatory response and promoting angiogenesis during the early stage of OA, respectively.^120^, ^121^ We speculate that aberrant GNL3 expression is associated with the expression and function of downstream mRNAs involved in the OA pathogenesis.

Although GNL3 has been proven to be associated with OA, these data were mainly based on bioinformatic analysis and cellular experiments.^120^ The GTPR mechanism of GNL3 in the OA pathogenesis should be clarified in the future for a better understanding of the bioprocess of chondrocytes during the development of OA and developing a potential therapeutic strategy for the treatment of OA.

### Cold‐inducible RNA‐binding protein

Cold‐inducible RNA‐binding protein (CIRBP) is a cold‐shock protein belonging to a member of the glycine‐rich RBPs family.^122^ CIRBP was first identified as a damage‐associated molecular pattern (DAMP) molecule, mediating cellular response to environmental stimuli, such as tissue injury and inflammation.^123^ CIRBP is induced by multiple external stimuli, including infection, hypoxia, hypothermia, and ultraviolet radiation, and it acts as a DAMP molecule that aggravates the inflammatory response.^124^, ^125^ Several studies have demonstrated that increased expression of CIRBP in the plasma predicts the poor prognosis of patients with sepsis.^126^, ^127^ In addition, it has been reported that increased expression of CIRBP can induce the expression of inflammatory cytokines by activating the NF‐kB and ERK signaling pathways.^128^, ^129^, ^130^ CIRBP might bind to its target mRNA at the post‐transcriptional level and affect the expression of its downstream inflammatory cytokines. Recent studies have shown that the expression of CIRBP in the synovial fluid is significantly increased in OA samples compared with normal controls.^43^, ^131^ This study suggests that increased expression of CIRBP in the synovial fluid might be an independent predictor of OA joint severity. We believe that CIRBP is not only an indicator but also a regulator during the development of OA. In addition, its PTGR mechanism should also be investigated and clarified in detail in the future.

### RBPMS

RBPMS belongs to the RRM family, including multiple alternatively spliced transcripts.^132^, ^133^ Earlier studies showed that RBPMS was exclusively expressed in the retinal ganglion cells (RGCs) and is a marker for RGCs.^134^, ^135^ Recently, RBPMS has also been reported to be expressed in the heart in vertebrates.^136^ RBPMS is involved in the PTGR mechanism of pre‐mRNA, including splicing, transport, sublocation, and stability.^135^ Nakagaki‐Silva *et al*. found that expression of RBPMS is positively associated with splicing events in vascular smooth muscle cells, and RBPMS controls other splicing factors, including MBNL1, MBNL2, LSM14B, and Myocardin.^137^ AS is believed to play an essential role in the regulation of gene diversity associated with the OA pathogenesis.^138^, ^139^ Sun et al. (2006) demonstrated that RBPMS binds to Smads and increases the expression of Smad2 and Smad3 by enhancing phosphorylation at the C‐terminal of Smad2 and Smad3.^140^ Smad2 and Smad3 are also involved in maintaining chondrocyte homeostasis through regulation of Col II and ACAN.^141^, ^142^ Shanmugaapriya et al. demonstrated that RBPMS interaction with Smads, increased TGF‐β‐mediated Smad2/3 transcriptional activity, and decreased RBPMS expression be involved in the OA pathogenesis.^44^ Further study should be conducted to investigate the specific role of RBPMS by overexpressing or inhibiting RBPMS in OA animal studies and develop a potential strategy for maintaining cartilage homeostasis in OA pathogenesis based on these data.

### RBMS3

Single‐stranded interacting protein 3 (RBMS3) is an RBP that belongs to the C‐myc gene single‐strand binding protein (MSSP) family.^143^ RBMS3 is a tumor suppressor gene, and downregulation of RBMS3 is correlated with poor prognosis in in various human cancers.^144^, ^145^ The suppressive effect of RBMS3 is mediated by a domain located at the 3p region of chromosome 3 that generates multiple tumor suppressor genes.^146^, ^147^ It is a favorable prognostic marker for many human cancers. RBMS3 has been reported to be involved in the promotion of fibrosis and the formation of RBMS3.^143^, ^148^ RBMS3 also regulates its target mRNAs at the post‐transcriptional level.^143^, ^148^, ^149^ Wang *et al*. (2020) found that increased expression of RBMS3 might exert a protective effect on IVD by inhibiting the Wnt/β‐catenin signaling pathway.^150^ The β‐catenin protein‐mediated Wnt/β‐catenin signaling pathway plays an important role in the regulation of NP cells metabolism.^151^ Wang et al. demonstrated that the overexpression of RBMS3 can promote the proliferation of NP cells and inhibit the degeneration of ECM by inhibiting the Wnt/β‐catenin signaling pathway.^150^ However, RBMS3 is a newly identified RBP in IVD. Its role in PTGR remains poorly understood, and research on RBMS3 in OA has not been reported.

## Potential therapeutic approaches for approaches for treating RNA binding proteins in ntervertebral disc degeneration and osteoarthritis

The etiology of IDD and OA is multifactorial and irreversible, arising from a complex interaction between predisposing genes and environmental factors.^52^, ^53^ A variety of surgical approaches have been conducted to treat patients with IDD and OA; however, these approaches are associated with postoperative morbidities and are expensive. Novel biological therapeutic agents should be highlighted and developed in the future to reverse the degenerative process of IDD and OA and prevent the development of chronic musculoskeletal disorders. Although several candidate anabolic agents have been proposed for therapy for IVD and cartilage regeneration,^152^, ^153^, ^154^ most of these therapeutic choices are being debated, and clinical outcomes are not definitive. Multiple RBPs have been reported to be involved in the pathogenesis of IDD and OA, suggesting that they are novel contributors to both diseases. With the accumulation of knowledge regarding the PTGR mechanism in IDD and OA, RNA‐based therapy is potentially a new approach to treat these degenerative musculoskeletal disorders. Therefore, these RBPs might serve as a new target for the management of IDD and OA.

### Antisense oligonucleotides

With the advancement of sequencing technologies, we can accurately identify new RBPs and their RNA targets in various disorders. Understanding their function and regulation in the pathogenesis of IDD and OA will help accelerate drug target discovery. Various RNA‐based technologies have also been applied for therapeutic purposes. Antisense oligonucleotides (ASOs) are highly modified synthetic RNA or DNA sequences designed to bind target RNA through complementary base pairing.^155^ ASOs are designed to exert their pharmacological effects through several mechanisms, including regulation of splicing events, inhibition of protein translation and degeneration of mRNA by recruiting RNase H.^155^, ^156^ Several ASO‐based drugs have already been applied to treat neurodegenerative disorders and cancers.^157^, ^158^, ^159^, ^160^ Spinal muscular dystrophy (SMA) is caused by *SMN1* mutations, leading to decreased SMN1 protein expression and impaired motor neuron function. Exon 7 on the *SMN2* gene is analogous to the SMN1 protein function.^161^, ^162^ Thus, ASO‐based drugs are designed to promote exon 7 by blocking the interaction of RBP hnRNP A1/A2 and the target RNA.^158^, ^159^ In addition, ASOs have also been applied to decrease the metastasis of lung cancer by inhibiting metastasis‐associated lung adenocarcinoma transcript 1.^160^ We speculate that ASO‐based drugs can also be designed to correct AS changes triggered by IDD or OA by modulating the binding of specific RBP and their target RNAs.

### Small interfering RNA

Fire et al. first explained that small interfering RNA (siRNA) could significantly reduce target mRNA expression compared to sense strand RNA and antisense strand RNA.^163^, ^164^ With the development of RNA technology, specially designed siRNA has been successfully applied to silence target mRNA.^163^ siRNA‐based drugs can effectively treat many diseases, including genetic disorders, cancer, and other diseases derived from gene overexpression.^165^ Recent studies have shown that overexpression of HuR can promote cellular growth in non‐small cell lung cancer (NSCLC) and enhance migration and invasion in vitro.^166^ HuR might be a potential therapeutic target for treating lung cancer. Numerous studies have found that complementary siRNA can exert therapeutic effects by downregulating the HuR expression in ovarian and lung cancer.^167^, ^168^ siRNA‐based drugs targeting HuR have been proven to inhibit migration and invasion of lung cancer cells by modulating interactions between HuR and pre‐mRNA and suppressing subsequent CXCR4, MMP‐2, and MMP‐9 expression.^169^ Although siRNA‐based drugs hold promise in cancer treatment, several barriers to delivering siRNA‐based drugs still need to be overcome. First, siRNA would be degraded in tissues by various nucleases in a few minutes.^170^ Second, the intrinsic “off‐target” effects caused by the uptake of siRNA‐based drugs by other cells or tissues can lead to adverse side effects.^171^ Significant efforts have been made to overcome these barriers. Efficient genome and chemical modification of siRNA‐based drugs have been performed to improve the stability of siRNA and reduce the “off‐target” effects.^163^

Taking a broad perspective, Antisense oligonucleotides and siRNAa has been considered as a promising treatment option for a variety of diseases. ASOs and siRNA‐based drugs have been extensively tested at different stages of clinical trials. Compared with protein‐based and small molecule drugs, RNA‐based therapeutics can be applicable for a wide range of targets, and their generation time and production cost are relatively low.^172^, ^173^ It appears to be an expansion in the scope of human disorders that can be treated with these approaches. It is expected that ASOs or siRNA‐based drugs targeting IDD and OA will be designed and applied in clinics in the near future. However, their efficiency and safety should be cautiously considered.

## Conclusion and Future Perspective

Although investigations of RBPs are still in their infancy, they have been identified as novel contributors to IDD and OA. Therefore, dysregulated RBPs might be promising diagnostic biomarkers and potential therapeutic targets. However, numerous challenges still need to be overcome. Although thousands of RBPs have been reported to be differentially expressed in various tissues, to our knowledge, only a few of them (six in IDD and six in OA) are proven to be involved in IDD or OA pathogenesis. It is widely recognized that ncRNA is a major target of RBPs and is also associated with PTGR in IDD and OA. Future studies should focus on the novel interaction between RBPs and ncRNAs during the onset and development of IDD and OD. Most studies indicated that RBPs exert their modulating function and reverse IDD or OA in cell experiments or rodent models. The molecular biology of IDD and OA in human beings is more complicated than in cell experiments or rodent models. More advanced technology and animal models should be established to clarify the regulation mechanism of RBPs in IDD and OA and precisely predict potential therapeutic targets. Further, more influencing factors should be considered.

RNA binding proteins in IVD or OA tissues do not act like those in other tissues or cells. These RBPs are tissue or cellular specific and they exert their role in specific situations upon external stimuli. Therefore, future study should be conducted to investigate the specific function of RBPs in IVD and OA tissues and reveal novel PTGR mechanisms during the development of IDD and OA.

RNA‐based drugs can be applied in an expanded scope of human disorders, including IDD and OA. However, since the function of RBPs are cellular and tissue specific, the same drug specially designed for target RBPs might exert distinct roles in the other tissues. In summary, PTGR by RBPs is implicated as a key mechanism in the pathogenesis of IDD and OA and has the potential for the development of novel therapeutic approaches for patients with IDD and OA.

## Author Contributions

WL, XL, and CX participated in the literature review. WL and YG designed the figure. WL wrote the draft of the review. WL, CX, and ZT polished the manuscript. WL and XL contributed equally to this work. All authors have read and approved the final manuscript.

## Conflict of Interest Statement

The authors declare that the research was conducted without any commercial or financial relationships that could be construed as a potential conflict of interest.

## Ethics Statement

Ethical approval dose not apply to this art.
